# Endoscopic Resection of Rectal Neuroendocrine Tumors: How Deep Should We Go?

**DOI:** 10.3390/jcm15114103

**Published:** 2026-05-26

**Authors:** Kasper Maryńczak, Przemysław Kasprzyk, Karol Pierzchała, Aleksandra Osielczak, Zofia Orzeszko, Łukasz Dziki, Michał Spychalski

**Affiliations:** 1 Department of General and Oncological Surgery, Medical University of Lodz, 90-419 Lodz, Poland; kasper.marynczak@umed.lodz.pl (K.M.); kasprzykprzemek@gmail.com (P.K.); aleksanda.osielczak@stud.umed.lodz.pl (A.O.); lukasz.dziki@umed.lodz.pl (Ł.D.); michal.spychalski@umed.lodz.pl (M.S.); 2 Department of Sleep Medicine and Metabolic Disorders, Medical University of Lodz, 92-215 Lodz, Poland; 3 Department of Surgery, Faculty of Health Sciences, Jagiellonian University Medical College, 31-501 Krakow, Poland

**Keywords:** rectal neuroendocrine tumor, endoscopic intermuscular dissection, endoscopic submucosal dissection, endoscopic resection, en bloc resection, neuroendocrine tumor

## Abstract

**Background:** Rectal neuroendocrine tumors (r-NETs) measuring 20 mm or less are increasingly diagnosed during colorectal cancer screening, but the optimal depth of endoscopic resection remains uncertain. Endoscopic submucosal dissection (ESD) is well established, whereas endoscopic intermuscular dissection (EID) may provide deeper resection for fibrotic or recurrent lesions. We hypothesized that EID would provide reliable deep-margin clearance without compromising safety. **Methods:** We retrospectively reviewed 42 consecutive patients treated at a tertiary center between 2018 and 2025. Thirty-two primary lesions underwent ESD and 10 lesions or scars suspicious for deep invasion underwent EID. Primary outcomes were en bloc and R0 resection; secondary outcomes were procedure time, adverse events, and length of stay. Groups were compared with the *t*, Mann–Whitney *U*, and chi-square tests. **Results:** En bloc resection was achieved in all cases. Histology confirmed R0 resection in all 26 primary lesions. Among 16 excision scars, 14 showed fibrosis only and 2 harbored grade 1 NET recurrence; both recurrent lesions were resected R0 with EID. Lesion size and procedure time were similar between groups. No major adverse events occurred. Self-limited intraprocedural bleeding occurred in five patients, and all patients were discharged within 2 postoperative days. **Conclusions:** Both techniques are safe and effective for r-NETs measuring 20 mm or less, and EID may be preferred for fibrotic or recurrent lesions. Large prospective multicentre studies are needed to validate the depth-tailored use of EID in r-NETs.

## 1. Introduction

Neuroendocrine tumors (NETs), once referred to as carcinoids, originate from disseminated neuroendocrine cells. Most NETs (70%) arise in the gastrointestinal tract, and the rectum is the second most common site after the small intestine [1]. Rectal neuroendocrine tumors (r-NETs) account for 12% to 27% of all NETs and 20% of gastrointestinal NETs [2]. They represent 1–2% of all rectal neoplasms, and their incidence has increased in recent years because of the wider use of colonoscopy and colorectal cancer screening [3]. The prevalence of r-NETs among participants in the Polish colorectal cancer screening program is estimated at 0.05–0.07% [4].

Rectal NETs are almost always asymptomatic and are incidentally detected during endoscopy [5]. Tumors below 1 cm, if well differentiated, usually have a low risk of metastasis, though the risk depends on Ki67 and lymphovascular invasion. NET classification into different degrees of malignancy (G1, G2, G3) is based on the proliferation index, the number of mitoses, and the degree of cell differentiation. G1 lesions are characterized by low potential, while G2, and especially G3, are tumors with high malignant potential and the risk of metastasis.

The treatment strategy for r-NET primarily depends on the size and depth of the tumor as well as the stage of metastasis. NETs smaller than 10 mm qualify for local resection without imaging diagnostics. For larger tumors, diagnostic imaging is necessary, and it is also advisable postoperatively in patients with high Ki-67 levels. The presence of metastases requires individualized multidisciplinary management depending on tumor burden, differentiation grade, resectability and patient status, including consideration of surgery, systemic therapy, and locoregional treatment. In the case of well-differentiated NETs smaller than 10 mm, endoscopic removal is recommended due to the low risk of local and distant invasion. Up to 90% of NETs are of this size, confined to the submucosa, and are well-differentiated; hence, endoscopic resection is considered the gold standard of therapy.

It is estimated that only 18% of lesions are accurately diagnosed among all neuroendocrine lesions endoscopically. Proper diagnosis of NETs before resection is associated with a higher rate of complete resection compared to their excision as polyps [6].

Tumors larger than 20 mm require surgical resection because of the high risk of distant spread and involvement of the muscularis propria. Tumors measuring 10–20 mm carry an intermediate risk of metastasis (5–15%), with a moderate risk of lymph node involvement, and endoscopic treatment can be challenging. The optimal approach remains debated [7]. According to the ENETS and UICC/AJCC guidelines, tumors measuring 10–19 mm require thorough assessment at first diagnosis to exclude invasion of the muscle layer and regional lymph nodes using rectal magnetic resonance imaging and/or rectal endoscopic ultrasound [8]. Some guidelines recommend local resection, especially for tumors up to 14 mm, whereas others favor radical surgery because of metastatic risk [9].

Endoscopic treatment options for NETs include polypectomy, endoscopic mucosal resection (EMR), and endoscopic submucosal dissection (ESD). EMR includes conventional EMR and modified techniques such as ligation-assisted EMR (L-EMR) and EMR after circumferential precutting (P-EMR). Conventional polypectomy is not recommended because of the low likelihood of R0 resection [10].

Endoscopic submucosal dissection (ESD) enables en bloc removal of larger lesions by dissecting the submucosal layer under direct endoscopic visualization. ESD is technically demanding, requires extensive training, and carries a higher risk of perioperative adverse events, most commonly bleeding and perforation, than EMR [11,12,13].

Studies comparing complete resection rates after ESD and EMR have reported mixed findings. Zhou et al. [13] reported a significantly higher proportion of R0 resection in the ESD group compared with EMR, with similar outcomes between ESD and modified EMR. Pan et al. [11] found L-EMR superior to ESD in R0 resection, with shorter procedure time and a lower risk of complications. Matsuhashi et al. [12] observed higher R0 rates and fewer recurrences after ESD than after modified EMR. Similarly, Yu et al. [14] reported that EMR and ESD were similarly safe and effective for tumors smaller than 10 mm, whereas ESD yielded a higher R0 resection rate for tumors measuring 10–20 mm. ESD also allows removal of larger lesions without invasive surgery [15].

Nevertheless, deep submucosal invasion can limit radical deep resection with ESD and is associated with a positive vertical margin [16,17]. According to several reports, endoscopic intermuscular dissection (EID), performed in the intermuscular plane, is a promising alternative, particularly in severe submucosal fibrosis with muscle retraction [18,19].

Studies suggest higher rates of complete resection with EID than with ESD while maintaining comparable safety and procedure time [16,17]. Recent retrospective cohort studies comparing EID and ESD have reported encouraging oncologic and safety outcomes, although available evidence remains limited [20,21]. Underwater EID may further improve visualization of the intermuscular plane and vascular structures, thereby facilitating smoother dissection and faster hemostasis [17].

However, further comparative studies are needed to establish the optimal endoscopic technique for NETs.

This study presents the results of endoscopic treatment of rectal neuroendocrine tumors at a specialized center with extensive experience in endoscopic submucosal dissection (an average of 250 procedures per year). In light of the growing interest in endoscopic intermuscular dissection and its particular relevance to rectal lesions, we analyzed our outcomes with both endoscopic submucosal dissection and endoscopic intermuscular dissection.

## 2. Materials and Methods

This retrospective cohort study included 42 consecutive patients (54.8% male; age range, 24–81 years) treated between 2018 and 2025. Most patients had complete baseline data for body habitus, with weight ranging from 53 to 125 kg, height from 154 to 186 cm, and body mass index from 20.05 to 43.25, as well as detailed comorbidity profiles. Three patients had missing data on weight, height, and body mass index, and four lacked documentation of lesion distance from the anal verge.

Within the study cohort, two distinct subgroups were identified: patients with primary lesions (n = 26; 61.9%) and those with secondary lesions (n = 16; 38.1%). Patients with secondary lesions had undergone endoscopic resection at outside institutions using conventional hot or cold snare polypectomy. Initial histopathological examinations demonstrated incomplete excision (R1) or indeterminate resection margins, and these patients were referred to our center for further treatment. The interval between the primary procedure and repeat endoscopic treatment ranged from 3 to 9 months.

Primary lesions were previously untreated yellowish submucosal tumors identified during colonoscopy, excised using the ESD or EID technique. Secondary lesions were post-resection scars after prior incomplete endoscopic excision (R1 confirmed histologically at outside institutions); qualification for repeat endoscopic resection was based on the endoscopic appearance of the scar and previous histopathological findings.

Routine preoperative endoscopic ultrasound (EUS) was not performed for r-NETs smaller than 10 mm in our center, as current evidence suggests limited additional diagnostic benefit in small lesions without endoscopic features of deep invasion [22,23]. In lesions exceeding 10 mm, additional staging with EUS and pelvic magnetic resonance imaging was performed to exclude muscularis propria involvement and locoregional lymph node metastases.

All patients underwent endoscopic excision, performed as endoscopic submucosal dissection in 76.2% of cases and as endoscopic intermuscular dissection in 23.8%. Lesions were located 2–12 cm from the anal verge. Knives used included Splash M-knife (DN-D2722B, Pentax Medical, Tokyo, Japan; 47.6%), DualKnife (J KD-655U, Olympus, Tokyo, Japan; 23.8%), FlushKnife (DK2620JI, Fujifilm Medical Co., Ltd., Tokyo, Japan; 16.7%), Goldknife (MK-T-1-235, Micro-Tech, Ann Arbor, MI, USA; 9.5%), and HybridKnife (I-Type I-Jet, ERBE, Tubingen, Germany; 4.0%).

**Figure 1:**
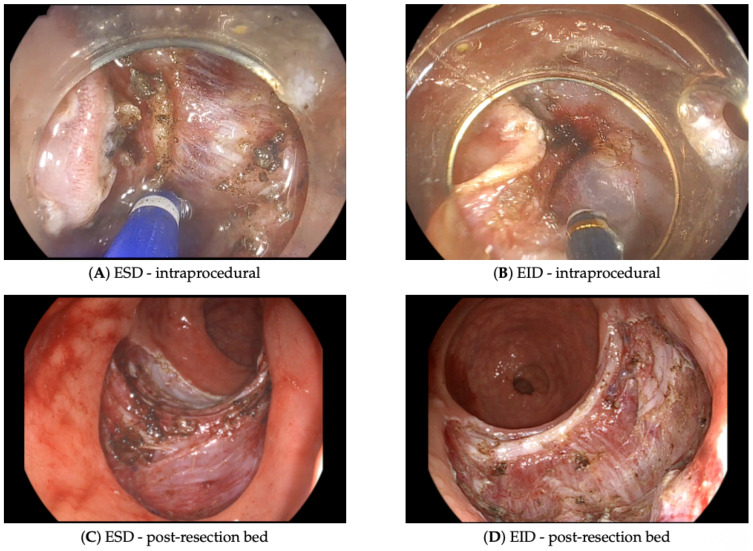
Endoscopic appearance of (**A**,**C**) endoscopic submucosal dissection (ESD) and (**B**,**D**) endoscopic intermuscular dissection (EID). Top row: intraprocedural views of the dissection plane—submucosal in ESD (**A**) and intermuscular, beneath the circular muscle layer, in EID (**B**). Bottom row: corresponding post-resection beds ((**C**), ESD; (**D**), EID), illustrating the deeper, intermuscular plane reached with EID compared with the submucosal plane of ESD.

In ESD, circumferential marking and submucosal injection are followed by mucosal incision and submucosal dissection beneath the lesion. In EID, after mucosal incision and partial submucosal dissection, the circular muscle layer is incised to access the intermuscular plane; dissection then proceeds in this plane to obtain a wider, deeper vertical margin. The main difference is depth: ESD remains in the submucosal plane, whereas EID extends into the intermuscular space, enabling deeper resection of fibrotic or recurrent lesions. EID was selected for lesions with suspected deep submucosal involvement, marked fibrosis, or recurrent/residual lesions after prior incomplete resection. Representative endoscopic images of both procedures, including the intraprocedural dissection phase and the post-resection bed, are shown in Figure 1.

All statistical analyses were performed using Python 3.9 with scipy, pandas, and numpy. Quantitative variables were assessed for normality with the Shapiro–Wilk test. Variables with normal distribution were presented as mean ± standard deviation and compared with the independent-samples *t* test. Variables with non-normal distribution were reported as median and interquartile range and compared with the Mann–Whitney *U* test. Categorical variables were expressed as counts and percentages and compared with the chi-square test. A two-sided *p* value < 0.05 was considered statistically significant. The statistical methods of this study were reviewed by Karol Pierzchała, Department of Sleep Medicine and Metabolic Disorders, Medical University of Lodz.

Baseline demographic, comorbidity, and procedural characteristics of the study cohort are summarized in Table 1.

## 3. Results

All lesions were resected en bloc, resulting in an en bloc resection rate of 100% in both the EID group (*n* = 10) and the ESD group (*n* = 32).

Median lesion size was 6.50 mm (interquartile range, 5.25–7.75) in the EID group and 5.00 mm (interquartile range, 4.75–8.25) in the ESD group (*p* = 0.640). The median width of the mucosal layer was 15.00 mm (interquartile range, 12.50–15.75) after EID and 20.00 mm (interquartile range, 12.00–22.00) after ESD (*p* = 0.086). The median deep margin measured 1.25 mm (interquartile range, 0.85–1.75) after EID and 0.50 mm (interquartile range, 0.20–0.90) after ESD (*p* = 0.147).

**Figure 2:**
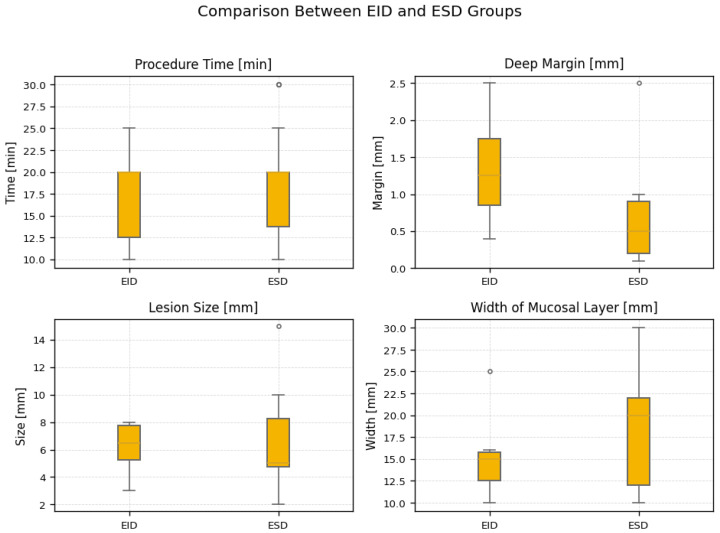
Box-and-whisker comparison of procedure time, deep margin, lesion size, and width of the mucosal layer between the EID and ESD groups. Boxes span the interquartile range with the median as the central line; whiskers extend to the most extreme non-outlier values, and open circles mark outliers. EID, endoscopic intermuscular dissection; ESD, endoscopic submucosal dissection.

Median procedure time was 20 min in both groups (*p* = 0.658), and no intraoperative complications were observed in either group. Figure 2 summarizes lesion size, deep margin, mucosal layer width, and procedure time for EID and ESD. Intraoperative parameters are detailed in Table 2.

Histopathological analysis separated patients into those with primary lesions and those with secondary lesions or scars after previous endoscopic procedures. Among patients with primary lesions, histopathology showed grade 1 neuroendocrine tumor and complete (R0) resection in all cases (*n* = 26). Among patients with secondary lesions (*n* = 16), 14 had scar tissue without residual tumor, whereas 2 had grade 1 neuroendocrine residual tumor in the scar. Endoscopic radicality (R0) was achieved in both cases (Table 3).

The postoperative course was uneventful overall, with discharge within 0–2 days and only one patient (2.4%) experiencing delayed discharge because of a non-ESD-related issue. No major complications occurred, although minor postoperative bleeding that required neither intervention nor pharmacologic treatment was observed in 11.9% of patients.

In summary, these findings indicate that both EID and ESD yield comparable outcomes regarding resection efficacy, procedure duration, and lesion characteristics. Overall, no significant differences were observed between the two methods, underscoring their similar effectiveness.

## 4. Discussion

Although rectal neuroendocrine tumors are often associated with an indolent histopathological course, their potential for local recurrence and rare metastasis remains clinically relevant.

In contrast to previously published comparative series, which were derived from heterogeneous colorectal lesions or focused on isolated technical aspects of EID, the present study addresses a specific and clinically defined population—rectal neuroendocrine tumors of 20 mm or less—and includes a substantial proportion of patients referred after incomplete resection performed at outside institutions. The originality of this work rests on three points. First, EID was applied selectively to fibrotic scars and recurrent r-NETs after incomplete prior resection, a subgroup that is rarely separated from primary lesions in the existing literature. Second, both ESD and EID were performed in a single high-volume centre with a uniform peri-procedural protocol, enabling a head-to-head technical comparison without inter-centre bias. Third, the analysis quantifies the deep margin—the parameter most directly relevant to the clinical question motivating the study (“how deep should we go?”)—rather than only en bloc and R0 rates. Even when interpreted together with the declared limitations (retrospective single-centre design, predominance of sub-10 mm lesions, modest EID sample size, and heterogeneous follow-up), these elements distinguish the present work from prior reports and support EID as a depth-tailored option within the endoscopic armamentarium for r-NETs.

Precise and complete removal of lesions is crucial for reducing the risk of further complications and improving prognosis. As noted above, a large proportion of rectal neuroendocrine tumors are smaller than 10 mm, which allows most patients to undergo minimally invasive endoscopic treatment, including ESD and modified EMR.

Endoscopic submucosal dissection enables en bloc resection with a high rate of complete removal (R0), which is essential in the treatment of r-NETs to minimize recurrence. Our findings are consistent with previous reports showing that ESD is highly effective and safe, with high en bloc resection rates and low complication rates. The particularly favorable outcomes observed in this series, including 100% en bloc resection and no major complications, may reflect the experience of our center and the limited sample size [24,25].

Traditional ESD may encounter difficulties in situations where the layers of the rectal wall are difficult to separate, which can result in incomplete resection. In such cases, endoscopic intermuscular dissection may be considered because it can achieve a greater deep margin and complete resection even when the lesion approaches the proper muscle layer. Although the present study did not show a statistically significant advantage over ESD, EID did not increase perioperative risk or procedure time and may therefore be a valuable alternative for larger tumors, fibrotic scars, or recurrent lesions.

Recent comparative studies have also demonstrated favorable outcomes of EID in rectal NETs. Han et al. reported higher vertical margin clearance with EID than with conventional ESD, while maintaining similar safety and procedure time; these findings are consistent with our observations, in which EID achieved numerically larger deep margins without increased adverse events [21]. Similarly, Yang et al. demonstrated that EID may improve complete vertical resection rates even in lesions smaller than 10 mm [20].

A particularly important subgroup comprises patients with lesions measuring 10–20 mm. In such cases, preoperative evaluation with magnetic resonance imaging and endoscopic ultrasound seems especially justified. This workup helps classify patients with an intermediate malignant risk and identify those who remain suitable for minimally invasive treatment. For such lesions, ESD and EID are attractive because lesion size itself is not a technical limitation of either method.

L-EMR is recommended for endoscopic treatment of rectal neuroendocrine tumors. Many studies report similar efficacy and safety for L-EMR and ESD, whereas some suggest that L-EMR may offer procedural advantages [26]. In the present study, the mean procedure time for both ESD and EID was 20 min.

Incomplete or recurrent lesions deserve particular attention. Risk factors for positive margins after endoscopic resection have been previously described and include submucosal invasion depth, fibrosis, and incomplete initial recognition of NET lesions [27]. Recognition of the neuroendocrine nature of rectal lesions during the initial colonoscopy remains low [6]. Consequently, r-NETs are often removed with a cold or hot snare or by conventional mucosectomy, techniques that are associated with high rates of incomplete reSection [10,28]. In our cohort of 16 previously treated cases, 14 scars contained no residual tumor despite an initial R1 histopathological result. One case showed obvious endoscopic recurrence that was confirmed histologically, whereas another scar looked benign endoscopically but contained grade 1 neuroendocrine tumor on histopathology; both were completely resected with EID. These findings suggest that endoscopic surveillance alone may be insufficient after incomplete resection and that referral to specialized centers for further endoscopic treatment should be considered.

Endoscopic hand suturing of the post-ESD defect has been proposed to secure the resection site and reduce complications. However, because most r-NETs are small and the overall safety profile of ESD and EID is favorable, routine use of endoscopic hand suturing is unlikely to improve safety meaningfully. Instead, it may prolong the procedure and increase technical complexity without clear additional benefit.

This study has some limitations. First, it was a retrospective single-center analysis with a relatively small sample size. Second, most lesions were smaller than 10 mm, limiting conclusions concerning larger rectal NETs with higher technical complexity. Third, the number of EID procedures was limited, and the follow-up duration was heterogeneous. Nevertheless, the study reflects real-world experience from a high-volume referral center with expertise in advanced endoscopic resection techniques, including management of recurrent and fibrotic lesions.

## 5. Conclusions

Both ESD and EID are safe and effective for endoscopic treatment of rectal neuroendocrine tumors measuring 20 mm or less, with 100% en bloc resection and comparable procedure times in this cohort. EID provides reliable deep-margin clearance in fibrotic or recurrent lesions and should be considered when submucosal invasion is suspected. Endoscopic surveillance alone appears insufficient after incomplete resection, and referral for further endoscopic treatment at specialized centers is advisable. Given the retrospective single-centre design and the modest number of EID procedures, the present findings should be regarded as hypothesis-generating; large prospective multicentre studies with standardised follow-up are needed to validate the depth-tailored use of EID and to define its role in current treatment algorithms for r-NETs.

## Figures and Tables

**Table 1:** Baseline demographic, clinical, procedural, and lesion characteristics of the study cohort. Unless otherwise indicated by an *n* annotation next to the variable, *n* = 42.

Variable	Category	*N* (%)/ Mean/Median	SD/IQR	Min	Max
*Demographics and anthropometrics*					
Age (years)		57.48 ^a^	13.78	24	81
Sex	Male	23 (54.76%)	–	–	–
	Female	19 (45.24%)	–	–	–
Weight (kg)		81.40 ^a^	15.44	53	125
Height (cm)		169.36 ^a^	8.49	154	186
Body mass index (kg/m^2^)		28.38 ^a^	5.12	20.05	43.25
*Comorbidities and medications*					
Nicotine dependence		4 (9.52%)	–	–	–
Arterial hypertension		20 (47.62%)	–	–	–
Diabetes mellitus		4 (9.52%)	–	–	–
Asthma or COPD		3 (7.14%)	–	–	–
Atrial fibrillation		2 (4.76%)	–	–	–
Other diseases		23 (54.76%)	–	–	–
Use of any medications		34 (80.95%)	–	–	–
Anticoagulants		2 (4.76%)	–	–	–
*Surgical history*					
Previous surgeries		28 (66.67%)	–	–	–
Abdominal surgeries		17 (40.48%)	–	–	–
Other surgeries		15 (35.71%)	–	–	–
Preoperative biopsy or polypectomy		37 (88.10%)	–	–	–
*Procedure and perioperative course*					
Procedure	ESD	32 (76.19%)	–	–	–
	EID	10 (23.81%)	–	–	–
Knife	Pentax M-knife	20 (47.62%)	–	–	–
	DualKnife	10 (23.81%)	–	–	–
	FlushKnife	7 (16.67%)	–	–	–
	Goldknife	4 (9.52%)	–	–	–
	HybridKnife	1 (2.38%)	–	–	–
Distance from anal verge (cm) (*n* = 38)		5.00 ^b^	5.00–8.00	2	12
Procedure time (min)		20.00 ^b^	11.25–20.00	10	30
En bloc resection		42 (100.00%)	–	–	–
Postoperative day of discharge		1.00 ^b^	1.00–1.00	0	2
Delayed discharge (non-ESD-related)		1 (2.38%)	–	–	–
Intraprocedural bleeding		5 (11.90%)	–	–	–
Endoscopic clip placement		5 (11.90%)	–	–	–
Coagulation		1 (2.38%)	–	–	–
Endoscopic hand suturing		1 (2.38%)	–	–	–
*Lesion and histopathology*					
Lesion status	Primary lesion	26 (61.90%)	–	–	–
	Scar	16 (38.10%)	–	–	–
Diagnosis	NET G1 (R0)	28 (66.67%)	–	–	–
	Scar without NET	14 (33.33%)	–	–	–
Scar outcome	Not applicable	26 (61.90%)	–	–	–
	Normal scar without NET	14 (33.33%)	–	–	–
	NET recurrence	2 (4.76%)	–	–	–
Lesion size (mm) (*n* = 26)		5.00 ^b^	5.00–8.00	2	15
Scar size (mm) (*n* = 4)		4.00 ^a^	1.41	2	5
Width of mucosal layer (mm) (*n* = 27)		17.33 ^a^	5.85	10	30
Deep margin (mm) (*n* = 15)		0.70 ^b^	0.30–1.00	0.1	2.5

For numerical variables, values are given as ^a^ mean with standard deviation or ^b^ median with interquartile range (Q1–Q3), together with minimum and maximum. For categorical variables, values are given as count and proportion of the corresponding category. Binary (yes/no) variables report only the count of patients with the condition present. ESD, endoscopic submucosal dissection; EID, endoscopic intermuscular dissection; COPD, chronic obstructive pulmonary disease; NET, neuroendocrine tumor; G1, grade 1; IQR, interquartile range; SD, standard deviation.

**Table 2:** Intraoperative parameters in the EID and ESD groups. Values are reported as median (interquartile range).

Variable	EID	ESD	*p* Value
Width of mucosal layer (mm)	15.00 (12.50–15.75)	20.00 (12.00–22.00)	0.086
Lesion size (mm)	6.50 (5.25–7.75)	5.00 (4.75–8.25)	0.640
Deep margin (mm)	1.25 (0.85–1.75)	0.50 (0.20–0.90)	0.147
Procedure time (min)	20.00 (12.50–20.00)	20.00 (13.75–20.00)	0.658

Sample sizes and overall ranges for each variable are reported in Table 1. EID, endoscopic intermuscular dissection; ESD, endoscopic submucosal dissection.

**Table 3:** Histopathological diagnoses and lesion characteristics in the EID and ESD groups.

Category	EID	ESD	*p* Value
*Diagnosis*			
NET G1 (R0)	6 (60.0%)	22 (68.8%)	0.71
Scar tissue only	4 (40.0%)	10 (31.3%)	
*Lesion type*			
Primary	6 (60.0%)	20 (62.5%)	1.00
Scar	4 (40.0%)	12 (37.5%)	
*Scar outcome*			
Not applicable	6 (60.0%)	20 (62.5%)	0.67
No recurrence	4 (40.0%)	10 (31.3%)	
NET recurrence	0 (0.0%)	2 (6.3%)	

EID, endoscopic intermuscular dissection; ESD, endoscopic submucosal dissection; NET, neuroendocrine tumor; G1, grade 1.

## Data Availability

The data that support the findings of this study are available from the corresponding author upon reasonable request.

## Abbreviations

The following abbreviations are used in this manuscript:

NET	Neuroendocrine tumor
r-NET	Rectal neuroendocrine tumor
ESD	Endoscopic submucosal dissection
EID	Endoscopic intermuscular dissection
EMR	Endoscopic mucosal resection
L-EMR	Ligation-assisted endoscopic mucosal resection
P-EMR	Endoscopic mucosal resection after circumferential precutting
ENETS	European Neuroendocrine Tumor Society
UICC	Union for International Cancer Control
AJCC	American Joint Committee on Cancer
MRI	Magnetic resonance imaging
Ki-67	Proliferation marker Ki-67
R0	Microscopically margin-negative resection
R1	Microscopically margin-positive resection
G1/G2/G3	Tumor grade 1/2/3

## References

[1] Dasari A., Shen C., Halperin D., Zhao B., Zhou S., Xu Y., Shih T., Yao J.C. (2017). Trends in the Incidence, Prevalence, and Survival Outcomes in Patients with Neuroendocrine Tumors in the United States. JAMA Oncol..

[2] Wang X.Y., Chai N.L., Linghu E.Q., Li H.K., Zhai Y.Q., Feng X.X., Zhang W.G., Zou J.L., Li L.S., Xiang J.Y. (2020). Efficacy and safety of hybrid endoscopic submucosal dissection compared with endoscopic submucosal dissection for rectal neuroendocrine tumors and risk factors associated with incomplete endoscopic resection. Ann. Transl. Med..

[3] Gallo C., Rossi R.E., Cavalcoli F., Barbaro F., Boškoski I., Invernizzi P., Massironi S. (2022). Rectal neuroendocrine tumors: Current advances in management, treatment, and surveillance. World J. Gastroenterol..

[4] Kaminski M., Polkowski M., Regula J. (2007). Prevalence and endoscopic features of rectal neuroendocrine tumors (carcinoids) among 50148 participants of the Polish colorectal-cancer screening programme. Gut.

[5] Moon C.M., Huh K.C., Jung S.A., Park D.I., Kim W.H., Jung H.M., Koh S.J., Kim J.O., Jung Y., Kim K.O. (2016). Long-Term Clinical Outcomes of Rectal Neuroendocrine Tumors According to the Pathologic Status After Initial Endoscopic Resection: A KASID Multicenter Study. Am. J. Gastroenterol..

[6] Fine C., Roquin G., Terrebonne E., Lecomte T., Coriat R., Do Cao C., de Mestier L., Coffin E., Cadiot G., Nicolli P. (2019). Endoscopic management of 345 small rectal neuroendocrine tumours: A national study from the French group of endocrine tumours (GTE). United Eur. Gastroenterol. J..

[7] Folkert I.W., Sinnamon A.J., Concors S.J., Bennett B.J., Fraker D.L., Mahmoud N.N., Metz D.C., Stashek K.M., Roses R.E. (2019). Grade is a Dominant Risk Factor for Metastasis in Patients with Rectal Neuroendocrine Tumors. Ann. Surg. Oncol..

[8] Fields A.C., McCarty J.C., Ma-Pak L., Lu P., Irani J., Goldberg J.E., Bleday R., Chan J., Melnitchouk N. (2018). New lymph node staging for rectal neuroendocrine tumors. J. Surg. Oncol..

[9] Ramage J., De Herder W., Delle Fave G., Ferolla P., Ferone D., Ito T., Ruszniewski P., Sundin A., Weber W., Zheng-Pei Z. (2016). ENETS Consensus Guidelines Update for Colorectal Neuroendocrine Neoplasms. Neuroendocrinology.

[10] Son H.J., Sohn D.K., Hong C.W., Han K.S., Kim B.C., Park J.W., Choi H.S., Chang H.J., Oh J.H. (2012). Factors associated with complete local excision of small rectal carcinoid tumor. Int. J. Color. Dis..

[11] Pan J., Zhang X., Shi Y., Pei Q. (2018). Endoscopic mucosal resection with suction vs. endoscopic submucosal dissection for small rectal neuroendocrine tumors: A meta-analysis. Scand. J. Gastroenterol..

[12] Matsuhashi N., Takahashi T., Tomita H., Araki H., Ibuka T., Tanaka K., Tanahashi T., Matsui S., Sasaki Y., Tanaka Y. (2017). Evaluation of treatment for rectal neuroendocrine tumors sized under 20 mm in comparison with the WHO 2010 guidelines. Mol. Clin. Oncol..

[13] Zhou X., Xie H., Xie L., Li J., Cao W., Fu W. (2014). Endoscopic resection therapies for rectal neuroendocrine tumors: A systematic review and meta-analysis. J. Gastroenterol. Hepatol..

[14] Yu Q., Zhang Y., Su Y., Zhao Q., Xiong K., Zhang L., Fang H. (2024). Optimization of Endoscopic Submucosal Dissection and Endoscopic Mucosal Resection Strategies for Rectal Neuroendocrine Tumors Within 20 mm. Am. Surg..

[15] Frydman A., Srirajaskanthan R. (2024). An Update on the Management of Rectal Neuroendocrine Neoplasms. Curr. Treat. Options Oncol..

[16] Sun P., Zheng T., Hu C., Gao T., Ding X. (2021). Comparison of endoscopic therapies for rectal neuroendocrine tumors: Endoscopic submucosal dissection with myectomy versus endoscopic submucosal dissection. Surg. Endosc..

[17] Liao S., Li B., Huang L., Qiu Q., Yang G., Ren J., Huang S. (2023). Endoscopic intermuscular dissection in the management of a rectal neuroendocrine tumor. Endoscopy.

[18] Dang H., Hardwick J.C., Boonstra J.J. (2022). Endoscopic intermuscular dissection with intermuscular tunneling for local resection of rectal cancer with deep submucosal invasion. VideoGIE.

[19] Rahni D., Toyonaga T., Ohara Y., Lombardo F., Baba S., Takihara H., Tanaka S., Kawara F., Azuma T. (2017). First reported case of per anal endoscopic myectomy (PAEM): A novel endoscopic technique for resection of lesions with severe fibrosis in the rectum. Endosc. Int. Open.

[20] Yang G., Huang S., Li B., Deng H., Liao S. (2025). Efficacy of endoscopic intermuscular dissection vs. endoscopic submucosal dissection in treating rectal neuroendocrine tumors <10 mm. Endosc. Int. Open.

[21] Han L., Hou H., Tan Y., Li R., Liang C., Lv L., Wang Y., Liu D., Min L. (2026). Endoscopic intermuscular dissection and endoscopic submucosal dissection for treatment of rectal neuroendocrine tumors: A retrospective cohort study. Minim. Invasive Ther. Allied Technol..

[22] Deprez P.H., Moons L.M.G., O’Toole D., Gincul R., Seicean A., Pimentel-Nunes P., Fernández Esparrach G., Polkowski M., Vieth M., Borbath I. (2022). Endoscopic management of subepithelial lesions including neuroendocrine neoplasms: European Society of Gastrointestinal Endoscopy (ESGE) Guideline. Endoscopy.

[23] Park S.B., Kim D.J., Kim H.W., Choi C.W., Kang D.H., Kim S.J., Nam H.S. (2017). Is endoscopic ultrasonography essential for endoscopic resection of small rectal neuroendocrine tumors?. World J. Gastroenterol..

[24] Spychalski M., Włodarczyk M., Winter K., Włodarczyk J., Dąbrowski I., Dziki A. (2021). Outcomes of 601 Colorectal Endoscopic Submucosal Dissections in a Single Western Center: Is Right Colon Location Still a Major Concern?. Surg. Laparosc. Endosc. Percutan. Tech..

[25] Spychalski M., Skulimowski A., Dziki A., Saito Y. (2017). Colorectal endoscopic submucosal dissection (ESD) in the West – when can satisfactory results be obtained? A single-operator learning curve analysis. Scand. J. Gastroenterol..

[26] Chen J., Ye J., Zheng X., Chen J. (2024). Endoscopic treatments for rectal neuroendocrine tumors: A systematic review and network meta-analysis. J. Gastrointest. Surg..

[27] Han L., Li J., Liang C., Chu Y., Wang Y., Lv L., Liu D., Tan Y. (2024). Risk factors for positive resection margins after endoscopic resection for gastrointestinal neuroendocrine tumors. Surg. Endosc..

[28] Binmoeller K.F. (2019). Underwater EMR without submucosal injection: Is less more?. Gastrointest. Endosc..

